# Grassland-to-crop conversion in agricultural landscapes has lasting impact on the trait diversity of bees

**DOI:** 10.1007/s10980-020-01141-2

**Published:** 2020-10-18

**Authors:** Gaëtane Le Provost, Isabelle Badenhausser, Cyrille Violle, Fabrice Requier, Marie D’Ottavio, Marilyn Roncoroni, Louis Gross, Nicolas Gross

**Affiliations:** 1 Centre d’Etudes Biologiques de Chizé UMR 7372, CNRS, Université de La Rochelle, 79360 Villiers en Bois, France; 2 INRAE, USC 1339, Centre d’Etudes Biologiques de Chizé UMR 7372, CNRS, Université de La Rochelle, 79360 Villiers en Bois, France; 3 LTSER « Zone Atelier Plaine & Val de Sèvre », Centre d’Etudes Biologiques de Chizé UMR 7372, CNRS, Université de La Rochelle, 79360 Villiers en Bois, France; 4 Senckenberg Biodiversity and Climate Research Centre SBIK-F, Senckenberg Gesellschaft für Naturforschung, 60325 Frankfurt, Germany; 5 INRAE, Unité de Recherche Pluridisciplinaire Prairies Plantes Fourragères, 86600 Lusignan, France; 6 UMR 5175 CEFE, Univ Montpellier, CNRS, EPHE, IRD, Univ Paul Valéry 3, 34293 Montpellier, France; 7 Université Paris-Saclay, CNRS, IRD, UMR Évolution, Génomes, Comportement et Écologie, 91198 Gif-sur-Yvette, France; 8 Laboratoire de Lutte Biologique, Département des sciences biologiques, Université du Québec à Montréal (UQAM), Succ. Centre-Ville, Montréal, QC C.P. 8888 Canada; 9 Université Clermont Auvergne, INRAE, VetAgro Sup, UMR Ecosystème Prairial, 63000 Clermont-Ferrand, France; 10 INRAE, UR 0633, URZF Unité de Recherche Zoologie Forestière, 45075 Orléans, France

**Keywords:** Functional trait diversity, Grassland, Habitat loss, Land-use changes, Landscape history, Plant–pollinator interactions

## Abstract

**Context:**

Global pollinator decline has motivated much research to understand the underlying mechanisms. Among the multiple pressures threatening pollinators, habitat loss has been suggested as a key-contributing factor. While habitat destruction is often associated with immediate negative impacts, pollinators can also exhibit delayed responses over time.

**Objectives:**

We used a trait-based approach to investigate how past and current land use at both local and landscape levels impact plant and wild bee communities in grasslands through a functional lens.

**Methods:**

We measured flower and bee morphological traits that mediate plant–bee trophic linkage in 66 grasslands. Using an extensive database of 20 years of land-use records, we tested the legacy effects of the landscape-level conversion of grassland to crop on flower and bee trait diversity.

**Results:**

Land-use history was a strong driver of flower and bee trait diversity in grasslands. Particularly, bee trait diversity was lower in landscapes where much of the land was converted from grassland to crop long ago. Bee trait diversity was also strongly driven by plant trait diversity computed with flower traits. However, this relationship was not observed in landscapes with a long history of grassland-to-crop conversion. The effects of land-use history on bee communities were as strong as those of current land use, such as grassland or mass-flowering crop cover in the landscape.

**Conclusions:**

Habitat loss that occurred long ago in agricultural landscapes alters the relationship between plants and bees over time. The retention of permanent grassland sanctuaries within intensive agricultural landscapes can offset bee decline.

**Electronic supplementary material:**

The online version of this article (10.1007/s10980-020-01141-2) contains supplementary material, which is available to authorized users.

## Introduction

The global decline of pollinators has motivated much research to better understand the underlying drivers (Goulson et al. 2015). Among the multiple pressures threatening bees, habitat loss has been suggested as a key contributing factor (Potts et al. 2010; Vanbergen et al. 2013). This is particularly true in European agricultural landscapes where the direct destruction of natural and semi-natural grasslands, through their conversion into annual crops over the last 50 years, has resulted in a reduction in nesting habitat and food resource availability for bees (Kremen et al. 2007). Assessing the direct and indirect impacts of habitat loss is therefore critical if we are to identify sound conservation strategies aiming at promoting pollinators in agricultural landscapes.

Plant and pollinator responses consecutive to habitat loss can be delayed through time (Bommarco et al. 2014). For instance, plants are expected to experience time-delayed responses to habitat loss because of long persistence times in seed banks (Purschke et al. 2014). Similarly, pollinators may exhibit delayed responses to local habitat loss through spillover from remnant habitats in the landscape (Bommarco et al. 2014). Such landscape processes can considerably slow down local species loss for years (Kuussaari et al. 2009). Yet, in the long run grassland-to-crop conversion can affect plant–pollinator mutualism by decreasing pollen dissemination (Aguilar et al. 2006) and threatening the persistence of bee-dependent plant in remnant grasslands (Clough et al. 2014). These time-delayed response to habitat loss constitutes an opportunity to avert further bee decline, if sound conservation and restoration efforts are implemented on time (Burkle and Alarcón 2011; Valiente-Banuet et al. 2015; Baude et al. 2016). Considering both current and long-term effects of land-use changes on plants and wild pollinators may therefore be essential to avoid irreversible bee loss and secure pollination services in agricultural landscapes (Larsen et al. 2005).

Understanding how habitat loss impacts mutualistic interactions and shape plant and bee communities is inherently challenging due to the high number of species and potential interactions at play (Bartomeus et al. 2016). Functional traits have been proposed as an efficient tool to overcome this challenge (Lavorel et al. 2013; Deraison et al. 2015; Gravel et al. 2016). The approach is based on the identification of traits involved in plant–bee interactions (i.e. ‘trait matching’, Bartomeus et al. 2016; Martin et al. 2019; Klumpers et al. 2019). For instance, tongue length in bees determines floral resource acquisition: bees with long tongue are expected to visit plants with deep corolla, suggesting a strong matching between flower and bee morphology (Bartomeus et al. 2016). Trait matching between plant and bee species can be scaled up at the community level (Suding et al. 2008). According to the trait matching between plant and bee species, the diversity of tongue length observed in bee communities should correlate positively with the diversity of floral resources available in local plant communities and in the surrounding landscape (Le Provost et al. 2017). Investigating how plant and bee trait diversity responds to habitat loss may allow inferring the importance of plant–pollinator interactions in shaping the functional structure of plant and bee communities (Bartomeus et al. 2016).

We used an extensive database of 20 years of field land-use records in an agricultural plain of 430 km^2^ situated in western France (Bretagnolle et al. 2018) to investigate the effects of current and past land use on plant and wild bee trait diversity in grasslands. Historically, the study region was a typical rural area composed of mixed crop-livestock systems (dairy goats and cows). Fifty years ago, grassland was the dominant land use, covering 80% of the study area. Since that time and up to the present day, shifts from livestock to annual crop production has resulted in a strong decline in grassland cover (grassland cover in 2014: 12% of the area) (Bretagnolle et al. 2018). Within the study area, we sampled 66 grassland fields with a wide range of land-use histories, while controlling for the effects of current landscape composition and the management of the sampled grassland field. Comparing grasslands in present-day landscapes having varying land-use change histories is an explicit way to quantify the legacy effect of land-use changes on plant and bee communities (Kuussaari et al. 2009; Bommarco et al. 2014). Land-use history was assessed by the age of the sampled grassland field (‘grassland age’) and a metric quantifying the time elapsed since grassland-to-crop conversion at the landscape level (‘grassland-to-crop conversion’). A high value of grassland-to-crop conversion indicates that most of the grasslands in the current landscape of the sampled grassland field were converted into annual crops long ago. We quantified plant and wild bee trait diversity in the focal grassland fields by measuring different morphological traits a priori involved in plant–bee interactions (see ‘Methods’ section). We tested the two following hypotheses: (i) according to the trait matching observed between plants and bees, bee trait diversity in the grassland fields is primarily determined by flower trait diversity of local plant communities, and increasing flower trait diversity of plant communities should increase the morphological diversity in bee communities; (ii) plants and their pollinators have time-delayed responses to grassland-to-crop conversion (i.e. might owe an ‘extinction debt’, *sensu* Kuussaari et al. 2009). Consequently, landscapes with recent grassland-to-crop conversion can still harbour diverse plant and bee communities, while their species pools might have strongly declined in historically crop-dominated landscapes, due to the scarcity of stable habitats (Bommarco et al. 2014). Additionally, specialist pollinator species characterized by a narrow feeding niche can suffer more than generalist species from habitat destruction (McKinney and Lockwood 1999). In landscapes where grasslands were converted into crops long ago, we thus expected that the functional diversity of bee communities may be reduced, mainly composed of generalist species, and that the trait matching between plants and bees will be less pronounced.

## Materials and methods

### Study area

The study was conducted in 2014 in the Long Term Socio-Ecological Research (LTSER) ‘Zone atelier Plaine et Val de Sèvre’ (ZAPVS) located in western France (Bretagnolle et al. 2018). The LTSER covered approximately 430 km^2^ of an intensively managed agricultural plain. In 2014, grasslands covered about 12% of the area and included artificial grasslands (i.e., alfalfa), temporary (sown with pure grasses or in mixtures with legume species) and permanent grasslands managed by grazing, mowing or abandoned. The remaining areas were covered by crops (66% of the surface), the main crops being straw cereal (34% of the surface), sunflower (10%), corn (9%) and oilseed rape (7%). Since 1994, land cover of the study area has been monitored on a yearly basis at the field level (~ 11,000 fields), by using about 30 land-use types (see Bretagnolle et al. 2018 for methodological details on land cover monitoring), and has been stored in a GIS database.

### Grassland selection

We monitored 66 grassland fields within the study area. The selected 66 grassland fields differed in local management practices and were selected among hay meadows with varying ages and vegetation types. The age of the grassland field was calculated as the time elapsed (counted in years) since the last tillage according to our land-use GIS database. The grassland age was set to 20 years when it has never been ploughed since 1994.

For each focal grassland field, we calculated the current landscape composition and a landscape metric linked to land-use history within a 1-km radius of the centre of the field (Fig. S1). The scale of 1 km is an adequate scale to describe landscapes relevant to bees as it has been shown that most foraging flights are within this distance (e.g. Holzschuh et al. 2007, 2016). For landscape composition, we considered landscape elements and metrics known to impact bees, i.e. the proportion of the landscape covered by grasslands, forests and mass-flowering crops (oilseed rape and sunflower) (Westphal et al. 2003; Bailey et al. 2014; Clough et al. 2014). For the land-use history metric, we characterized the time elapsed since grasslands had been converted for the first time into annual crops averaged at the landscape-level, i.e. the grassland-to-crop conversion. To do so, we first calculated the time (in years, starting from 1994) elapsed since the first grassland-to-crop conversion for all fields in a 1 km-radius surrounding each of the focal grassland fields. For instance, a field cultivated as annual crop since 1994 had a value of 20 years; a grassland field converted to crop in 2000 had a value of 14 years; a grassland field set in place from 1994 to 2014 had a value of 0. To account for the field size, this metric was weighted by the field area. We then averaged all field values at the landscape level. Thus, a high value of the grassland-to-crop conversion metric indicates that grasslands in the 1-km radius were converted to crops long ago. In the study area, sown grasslands are often included in crop rotation. As they are regularly ploughed, the conversion from sown grassland to crop was not explicitly considered in our metric.

At the end of the selection process, the 66 selected grassland fields included pure legume (alfalfa or clover) grasslands (*n* = 11), meadows sown with pure (e.g. ryegrass) (*n* = 4) or mixed grasses (*n* = 18), sown with legume and grass mixtures (*n* = 23), and spontaneous flora (*n* = 10). All grasslands were either temporary (i.e. grasslands with an age ≤ 5 years) (*n* = 34) or permanent grasslands (defined as grasslands with an age > 5 years) (*n* = 32), and their average age was 8 year-old (*sd* = 6.55). Current landscape composition metrics of the 66 1-km radius landscapes ranged from 0 to 35% for grassland, 0 to 32% for forest and 0 to 44% for mass-flowering crop cover. Note that the age of focal grassland fields, current landscape composition metrics and grassland-to-crop conversion metric were not correlated (Table S1).

### Plant survey and floral traits

A botanical survey was conducted in summer 2014 from the 4th to the 19th of August 2014. We used 10 quadrats of 1 m^2^ located randomly within each grassland field to estimate plant diversity and species abundance. In each quadrat, the number of species was recorded and a percentage cover of the quadrat surface was visually estimated for each plant species. Relative abundance per species was then calculated as the sum of the species cover in the 10 quadrats divided by the total cover of all species. The sampling effort was sufficient to properly estimate plant species richness in the studied grasslands (Fig. S2).

To characterize flower diversity, we focused on flower traits relevant in determining plant–bee interactions (Fontaine et al. 2006; Bartomeus et al. 2016). We measured flower traits linked to resource accessibility: the flower opening angle (A, °) measured at the widest part of the corolla; the corolla depth (Cd, mm); the diameter (Cw, mm) of one flower measured at the widest part of the corolla; the mean width (Cwm, mm) of one flower calculated as the mean of three measures of the corolla width; the distance from the nectaries to the anthers (NAD, mm); the nectar access (Na) defined as the ratio between corolla width and the distance from the nectaries to the anthers. We also measured traits that influence flower-handling strategies by bees (Spaethe et al. 2001): the ‘landing zone’ (LZ, mm), which corresponds to the diameter of the inflorescence or of the flower; and the number of flowers (Nf) in one inflorescence (the number of flowers was one for simple flower). Flower traits were measured on the 25 plant species secreting nectar that represented 80% of the total cover of plant species with nectar (Table S2). Five individuals per species were measured, and a mean trait value was calculated for each trait on the 25 plant species. All measurements were performed using a stereo microscope (Leica Microsystems M50) equipped with an integrated high definition microscope camera (Leica IC80 HD).

### Bee sampling and bee morphological traits

We sampled wild bees in each of the 66 grassland fields using coloured pan traps. This is a common method to estimate pollinator diversity in agricultural landscapes (e.g. Westphal et al. 2008; Davis et al. 2018; Sirami et al. 2019). Six coloured pan traps per grassland field were left in place for four consecutive days. One pan trap consisted in two different plastic cups (15 cm diameter filled with water and a few drops of liquid soap) treated with yellow, blue or white UV-reflecting paint. We used all colour combination in order to sample a good representation of the diversity of bee communities (Bukovinszky et al. 2017; Hall 2018). Sampling was carried out along two parallel 50-m transects, one located at the field edge (three pan traps consisting in the three combinations of two colours), the other 25 m away inside the field (three pan traps). The pan traps were located at each end and in the centre of both transects. The sampling was carried out in late summer 2014 (between the 4th and the 25th of August), during the peak period of wild bee diversity in this study area (Rollin et al. 2015), and when bees mainly forage on herbaceous plant species in semi-natural habitats (Rollin et al. 2013; Requier et al. 2019a, b). Catches of the six pan traps per grassland field were pooled, and the bees were preserved in ethanol. Although the Western honey bee *Apis mellifera* can present a dual nature as managed and wild species in Western Europe (Requier et al. 2019a, b), we excluded *A. mellifera* from the samples given that managed colonies are substantially dominant in the study area (*pers. obs.* F. Requier). Thus, we used the term ‘bees’ as a surrogate for ‘wild bees’. The sampling effort was sufficient to properly estimate bee species richness in the studied grasslands (Fig. S2).

We measured bee traits related to body size, foraging and resource acquisition. Body size is a trait associated to foraging distance (Greenleaf et al. 2007), and pollination efficiency (Larsen et al. 2005). Foraging distance can also be influenced by wing size (Foster and Cartar 2011). Bee mouthparts were measured as they determine bee efficiency in acquiring floral resources (Klumpers et al. 2019) and of plant–bee network structure (Bartomeus et al. 2016). Body size (BS, mm) was measured as the inter-tegular distance, i.e. the distance between the wing bases (Greenleaf et al. 2007; Forrest et al. 2015). We also measured body length (Bl, mm), wing area (Wg, mm) and wing length vs. body size ratio (Wg:BS). Finally, we measured different parts of bee head and proboscis: the head width (Hw, mm), the prementum length (Pl, mm) and the glossa length (Gl, mm). We calculated the ratio between head width and body size (Hw:BS), between prementum length and body size (Prl:BS) and between glossa length and body size (Gl:BS) (Bartomeus et al. 2016; Cariveau et al. 2016). Measurements were performed using the stereo microscope (Leica Microsystems M50) equipped with an integrated high definition microscope camera (Leica IC80 HD).

Bee morphological traits were measured directly on the trapped individuals. The mean number of individual bees captured per grassland field over the six pan traps was 49 individuals. Traits were measured on 30 randomly selected individuals within each bee community (or all individuals when the number of captured bees was < 30, which was the case in 42% of the sampled grassland fields). Bees were identified at the species level. In total, 1050 individuals were measured, belonging to 60 wild bee species representative of the most abundant species in the study area (see Rollin et al. 2015, Table S3 and Fig. S2). Measuring all species in all communities—including the very rare species—is challenging and not necessarily needed to properly estimate functional diversity as the selected indices of functional diversity used in our study are not influenced by very rare species (Blonder et al. 2014).

### Statistical analyses

All statistical analyses were performed using the R statistical software (R Development Core Team 2016) version 3.3.1.

#### Trait-based characterization of plant and bee communities

We performed two principal component analyses (PCA) on flower traits, and on bee traits at the individual level to evaluate the correlations between the different traits measured, and to identify major functional dimensions (Devictor et al. 2010). We used the *varimax* procedure to maximize the correlations between PCA axes and traits. Based on the PCA, we selected three independent floral traits, each being highly correlated with one PCA axis (Fig. S3a and b): the flower width (i.e. the corolla diameter), the flower depth (i.e. the corolla depth) and the landing zone. For bees, we selected three independent bee traits, each being highly correlated with one PCA axis (Fig. S3c and d): body size; glossa length, a key trait involved in plant–bee mutualism (Bartomeus et al. 2016); and the prementum length vs. body size ratio, a trait that can determine bee efficiency in acquiring floral resources (Borrell 2005; Klumpers et al. 2019).

We then characterized the multi-trait functional diversity of plant and bee communities in each of the surveyed grasslands by calculating the volume of the multi-trait space occupied by all species (for plants) or individuals (for bees) belonging to a grassland field, using the R *hypervolume* package (Blonder et al. 2014, 2018). Contrary to other multidimensional functional diversity metrics (e.g. Cornwell et al. 2006), the hypervolume takes into account holes in the phenotypic space, giving a more accurate approximation of the functional space occupied by a community (Blonder et al. 2014). In addition, we calculated plant community mean trait values for each selected floral trait as the average of trait values in a given grassland, weighted by species abundance (following Garnier et al. 2004).

As there is an intrinsic mathematical link between the hypervolume estimates and the number of species or individuals recorded in the community, we randomly sampled the same number of species or individuals in each plant and bee community, respectively, using a rarefaction technique. This number was equal to that of the community with the lowest number of species or individuals (i.e. five). We then estimated the hypervolume on this subset, with constant number of species or individuals, and repeated the procedure 100 times for each community. The trait diversity was calculated as the mean hypervolume over all the 100 random samples. Note that the raw hypervolumes—calculated directly with all species or individuals sampled in the grassland—were highly correlated (r ≥ 0.96) to the hypervolumes corrected for the number of species recorded or individuals trapped (Fig. S4).

#### Effects of land-use on plant communities

We used linear models to evaluate the effects of current and past land use on the trait diversity of plant communities, i.e. floral hypervolume. We included in our models the effects of grassland field age and of the metric of grassland-to-crop conversion at the landscape scale. We also included current landscape composition metrics (% grassland, % forest and % mass-flowering crop areas). We used plant productivity as a proxy for grassland management intensity, as it is related to fertilizer inputs. This was assessed by harvesting plant biomass every month (between February and August 2014) above a cutting height of 5 cm from the soil surface, within five 35 × 35 cm quadrats per grassland. Grassland productivity was then calculated as the weight of dried plant material (oven-dried at 60 °C for 72 h) product per square meter per day between the initial biomass measurement and peak biomass (end of May). To account for potential non-linear effects, we considered quadratic terms for current land-use variables and land-use history. Finally, our model integrated latitude and longitude to correct for additional spatial effects not accounted for by the local and landscape predictors (see correlations among the predictors in Table S1). We performed model simplification using a backward regression procedure in the R *MASS* package (Venables and Ripley 2002), and we further kept the models with lower AICc (Δ AICc < 2). If multiple models were selected within a Δ AICc < 2, a model averaging procedure was performed rather than focusing on the model with the best AICc, in order to determine average parameter coefficients for the best final set of predictors (García-Palacios et al. 2018; Le Bagousse-Pinguet et al. 2019; Sirami et al. 2019). We used the *MuMIn* package (Bartoń 2014) and the *dredge* function that provides an average value over the selected models for the best predictors. Model residuals were inspected for constant variance and normality. We standardized all variables (*z*-scored: mean-centred and divided by the standard deviation) to interpret parameter estimates on a comparable scale (Schielzeth 2010). We calculated the relative effects of the parameter estimates for each set of predictors to evaluate the relative importance of each predictor on flower trait diversity of the plant community. This method is similar to a variance decomposition analysis since we *z*-scored all predictors.

#### Effects of land-use on bee communities and on the relationship between flower and bee morphological diversities

We included in the model the functional properties of the flower community (community weighted means of the selected traits, and floral hypervolume) as predictors of bee trait diversity. To test how land use impacts the interactions between plants and bees, we included in the model the same land use factors as for the analysis of flower trait diversity. We added in the model two-way interactions between current land use, land-use history and the flower trait diversity. Finally, as for the analysis of flower trait diversity, our model integrated latitude and longitude, and quadratic terms for current land-use variables and land-use history. We used the same analytic procedure described above to select the best set of predictors for bee morphological diversity.

To illustrate the effects of typical land-use changes that occurred in agricultural landscapes in Western Europe on the trait diversity of bee communities, we computed bee hypervolume in a focal grassland for three situations that resulted from contrasted past and present land-use management: (i) a current situation that corresponds to landscapes with low land-use intensification, similar to those observed twenty years ago in the study region, i.e. a permanent focal grassland (~ 15 year-old) with diverse plant communities, situated in a landscape with recent grassland-to-crop conversion (5 years) and where the landscape was currently composed of 9% of mass-flowering crops and 20% of permanent grasslands; (ii) a situation that corresponds to moderately intensified agricultural landscapes, i.e. a permanent focal grassland (~ 15 year-old) with diverse plant communities, situated in a historically crop-dominated landscape (grassland-to-crop conversion of 10 years on average) where only few permanent and plant species rich grasslands have been maintained (e.g. through agri-environmental scheme subsidies), and currently composed of 15% of mass-flowering crops and 10% of permanent grasslands; (iii) a situation that corresponds to intensive agricultural landscapes observed currently in the study area, i.e. a temporary grassland (< 5 year-old) with low flower trait diversity, situated in a crop-dominated landscape (grassland-to-crop conversion of at least 20 years), and currently composed of 25% of mass-flowering and 8% of species-poor temporary grasslands. Bee hypervolumes were calculated by gathering individuals from different sampled grasslands corresponding to each category. We then randomly sampled 100 individuals from each pool and estimated the bee hypervolume on this subset. The procedure was repeated 100 times for the three categories and the three hypervolumes were calculated as the mean hypervolume over the 100 random samples.

## Results

**Fig. 1:**
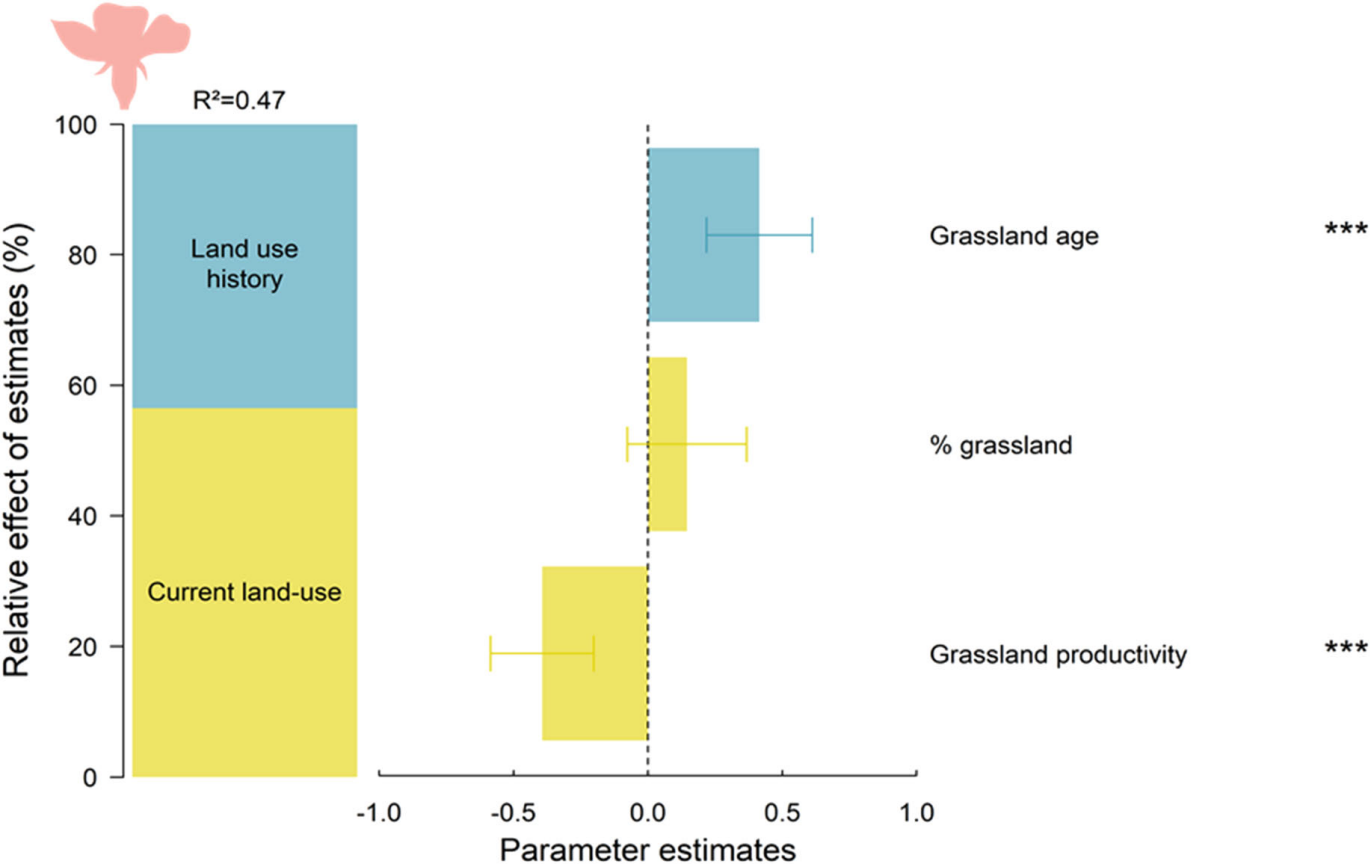
Relative effects of estimates (%) of each group of predictors (i.e. land-use history and current land use) and parameter estimates (± SE) resulting from the model averaging procedure on the flower trait diversity of plant community (hypervolume, sd^3^). P-values of the best selected models for each model parameter are given, *P-value < 0.05; **P-value < 0.01; ***P-value < 0.001. We considered only plants with nectar in the analyses. The flower trait diversity was log-transformed and all variables were scaled. See also Table S6 for details on model selection and Table S7

Past and current land-use had significant effects on the flower trait diversity of plant communities (i.e. floral hypervolume), with an *R*^2^ = 0.47 (Fig. 1; see also Table S6 for AICc-based model selection and Table S7). The effect of past land use was determined by a positive effect of the sampled grassland age (57% of explained variance, P-value < 0.001), where flower trait diversity within plant communities was higher in old permanent grasslands. The effect of current land use was mainly due to a negative effect of the grassland productivity (43% explained variance, P-value < 0.001) (Fig. 1).

**Fig. 2:**
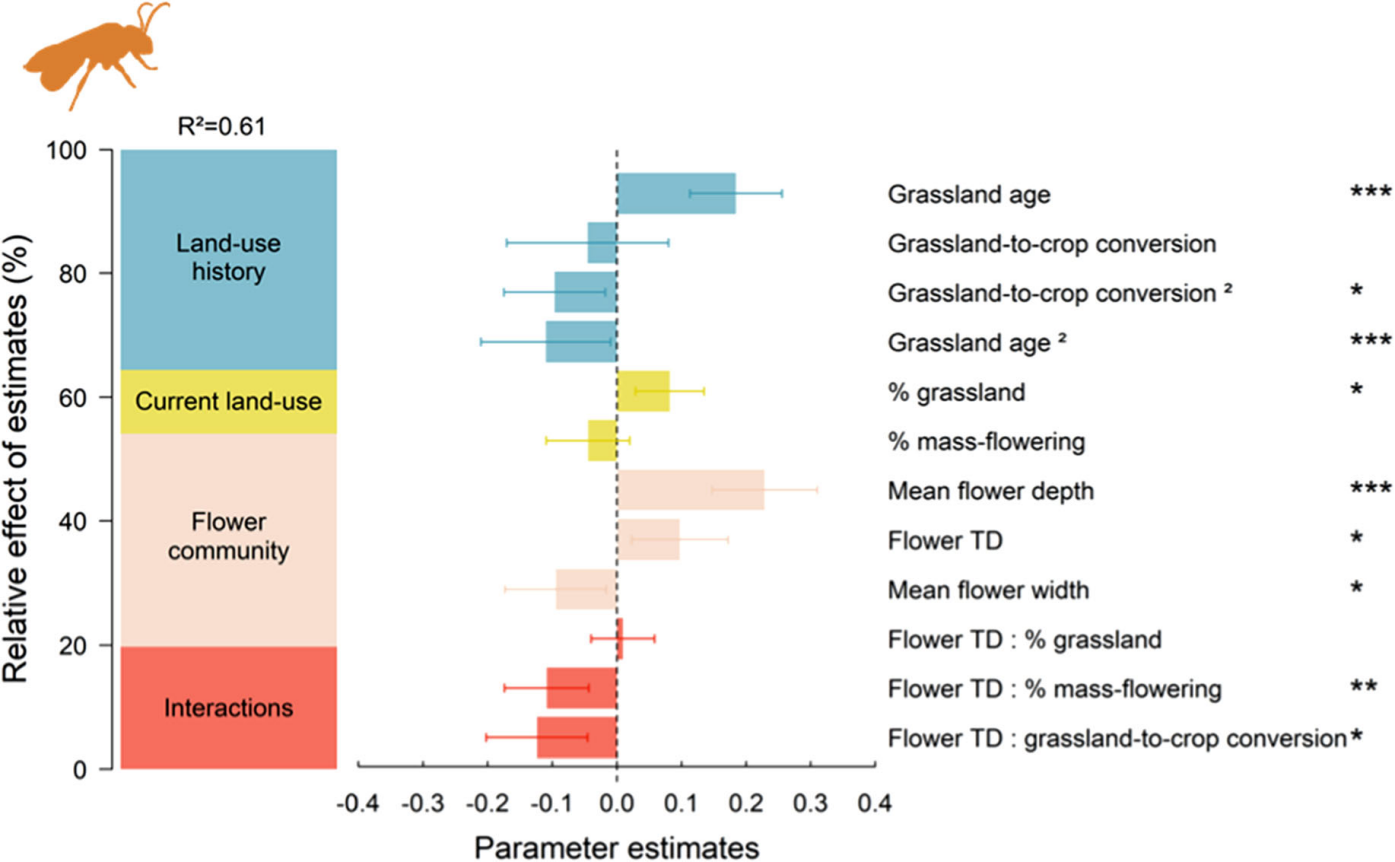
Relative effects of estimates (%) of each group of predictors (i.e. land-use history, current land use and flower community) and their interactions on bee trait diversity (hypervolume, sd^3^) and parameter estimates (± SE) resulting from the model averaging procedure. ‘Flower TD’ is the abbreviation for ‘Flower trait diversity’. P-values of the best selected models for each model parameter are given, *P-value < 0.05; **P-value < 0.01; ***P-value < 0.001. Bee trait diversity and flower trait diversity (hypervolumes, sd^3^) were log-transformed and all variables were scaled. See also Table S8 for details on model selection and Table S9

Bee trait diversity (i.e. bee hypervolume) was largely explained by the flower trait diversity (i.e., floral hypervolume) and individual floral trait values (altogether 34% of explained variance) (Fig. 2; see also Table S8 and Table S9). Specifically, the flower trait diversity had a positive effect on bee trait diversity (8% of explained variance, without interactions, P-value = 0.012), indicating that functionally diverse plant communities support diverse bee communities. The mean flower depth had a positive effect on bee hypervolume (19% of explained variance, P-value < 0.001), while the mean flower width had a negative effect (8% of explained variance, P-value = 0.021), suggesting that plant communities dominated by flowers with narrow and deep corollas are positively associated with functionally diverse bee communities.

**Fig. 3:**
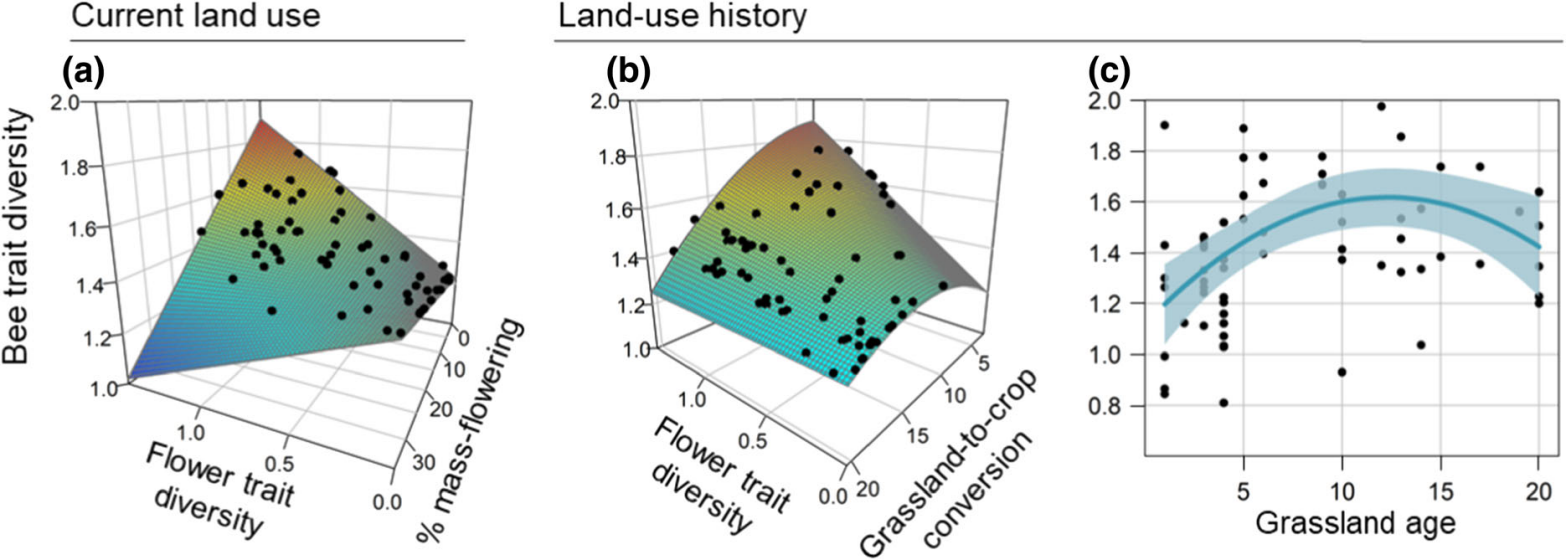
Significant effects of **a** flower trait diversity interacting with the percentage of mass-flowering crops in the landscape; **b** flower trait diversity interacting with grassland-to-crop conversion, and **c** age of the grassland on bee trait diversity. The colour gradient in panels **a** and **b** corresponds to bee trait diversity values, ranging from low values (blue) to high values (red). Black dots show the overall gradients in flower trait diversity, the percentage of mass-flowering crops in the landscape, the landscape grassland-to-crop conversion and the grassland age for the 66 grasslands. The shaded area in panel **c** indicates the 95% confidence intervals. Bee trait diversity and flower trait diversity (hypervolumes, sd^3^) were scaled and log-transformed

The positive relationship between flower and bee trait diversity was modulated by current landscape composition and land-use history (Figs. 2 and 3). Bee trait diversity was explained by two significant interactions between flower trait diversity and: (i) the proportion of mass-flowering crops in the surrounding landscape (9% of explained variance, P-value = 0.001); (ii) the grassland-to-crop conversion (10% of explained variance, P-value = 0.013). We found a strong positive relationship between flower and bee trait diversities in landscapes with a low mass-flowering crop cover (Fig. 3a) and recent grassland-to-crop conversion (Fig. 3b). In contrast, this relationship weakened in landscapes dominated by mass-flowering crops or where grasslands have been converted to arable fields for a long time.

At the local scale, grassland age had a quadratic effect on bee trait diversity (24% of explained variance, P*-*value < 0.001) which was higher in 10 to 15 year-old grasslands (Fig. 3c). Finally, current landscape composition variables accounted for 10% of explained variance, mostly due to a positive effect of the proportion of grasslands in the surrounding landscape (7% of explained variance, P-value = 0.027) (Fig. 2).

**Fig. 4:**
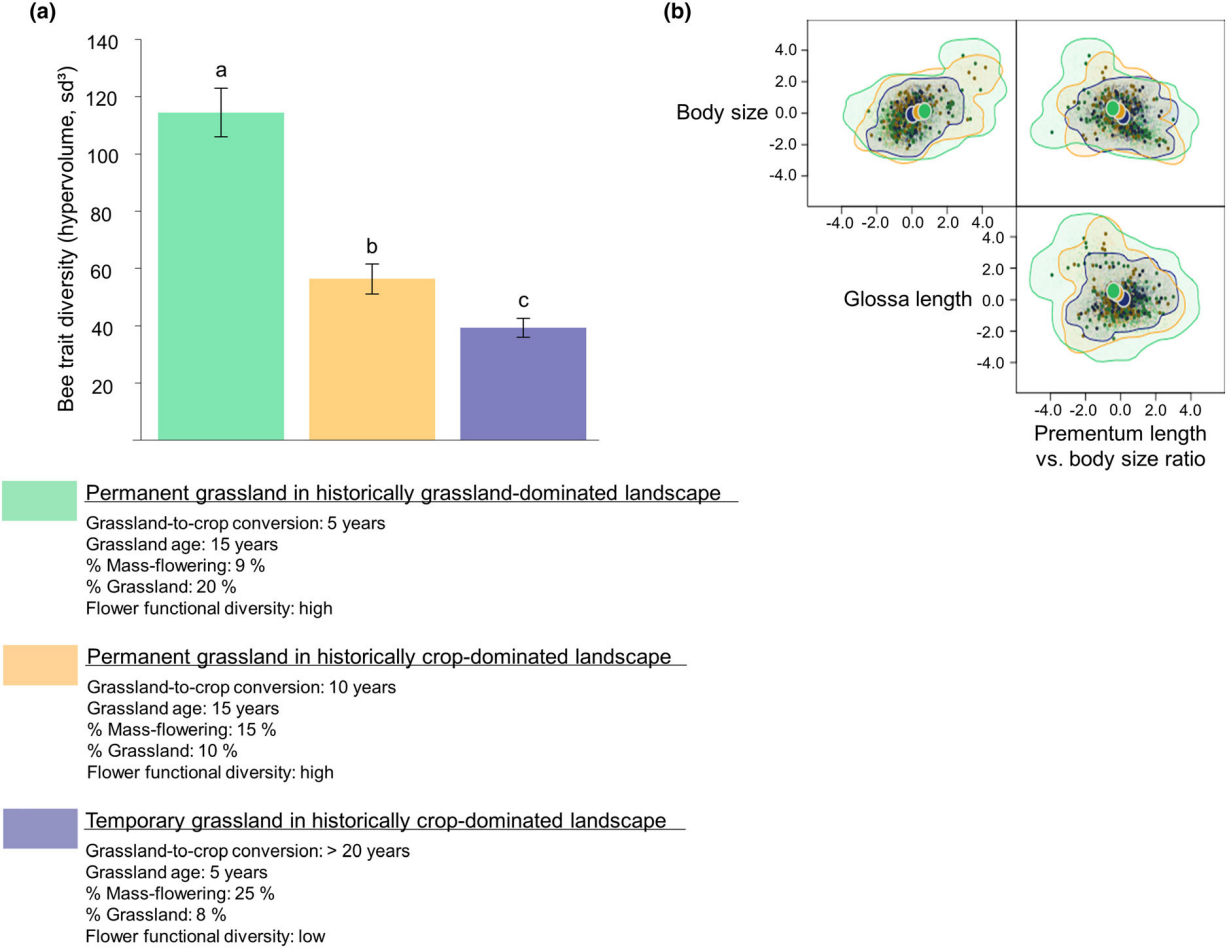
Observed bee trait diversity calculated for grasslands with contrasted floral diversity and landscape land-use history: (i) permanent grasslands with diverse plant communities in a historically grassland-dominated landscape, in green (sampled grasslands were around 15 year-old with high flower trait diversity, situated in a landscape where grasslands were converted into annual crops 5 years ago, 9% of mass-flowering crops, 20% of grasslands); (ii) permanent grasslands with diverse plant communities in a crop-dominated landscape with few remnant permanent grasslands, in orange (sampled grasslands were around 15 year-old with high flower trait diversity, situated in a landscape where grasslands were converted into annual crops 10 years ago, 15% of mass-flowering crops and 10% of grasslands); (iii) temporary grasslands with low flower diversity, situated in a crop-dominated landscape where only temporary grasslands remain, in blue (sampled grasslands were around 5 year-old with low flower trait diversity, situated in a landscape where grasslands were converted into annual crops at least 20 years ago, 25% of mass-flowering crops and 8% of grasslands;). (a) Bee trait diversity (hypervolume, sd^3^) observed in each category. Significant differences are indicated with letters. (b) Pairwise plots representing the hypervolume for each category, along traits (scaled) of the three-hypervolume dimensions: body size, glossa length and prementum length vs. body size ratio. The coloured lines are the contour lines of each hypervolume. The small coloured dots are the uniformly random samples from the inferred hypervolume. The large, filled, and coloured circles are the hypervolume centroids

When evaluating how contrasted land-use managements (combining focal grassland age, grassland cover in the current landscape and the history of grassland-to-crop conversion) affected the trait diversity of bee communities, we found that bee trait diversity strongly decreased in historically crop-dominated landscapes where grassland-to-crop conversion occurred long ago, compared to landscapes where grassland-to-crop conversion occurred only recently. Bee trait diversity in permanent grassland with high flower trait diversity decreased by half when the focal grassland was situated in a historically crop-dominated landscape compared to when it was situated in a landscape recently converted to crop. In the case of a temporary grassland with low flower trait diversity and situated in historically crop-dominated landscape with few temporary grasslands, bee trait diversity decreased by a factor of three compared to a species-rich permanent grassland situated in a historically grassland-dominated landscape (Fig. 4).

## Discussion

Our study shows that past grassland-to-crop conversion can have lasting negative impacts on present-day bee trait diversity. We found that increasing flower trait diversity increased bee trait diversity in grasslands (Fig. 2), highlighting the matching between the multidimensional morphological diversity of plant communities and their pollinators. However, the positive relationship between plant and bee trait diversities disappeared in agricultural landscapes where much of the land was converted into annual crops long ago, compared to similar current landscapes but with more recent grassland-to-crop conversion. Hence, our results suggest that past land-use changes can alter plant–bee interactions over time threatening their long-term persistence in agricultural landscapes.

### Plant–bee trait matching explains bee trait diversity

Although clear matching between some plant and bee traits has been identified (Bartomeus et al. 2016; Klumpers et al. 2019), quantifying how plant–bee trait matching influences the whole bee communities has been challenging so far. Using a multi-trait approach, we found that bee trait diversity increased with increasing flower trait diversity in grasslands (Fig. 2). This result highlights a strong matching between the multidimensional phenotypes of plants and their pollinators. It supports the idea that flower and bee morphological traits interact in complex ways to determine the diversity and abundances of bees at the community level (Coutinho et al. 2018).

In addition to flower diversity, flower mean traits of the plant communities were important parameters to quantify the net effect of flower trait diversity on bee communities. For instance, we found that the community mean flower depth had a positive effect on bee trait diversity (Fig. 2). Previous studies demonstrated that pollinators with long mouthparts forage preferentially on narrow or tubular flowers (Fontaine et al. 2006; Campbell et al. 2014; Klumpers et al. 2019), while short mouthpart pollinators could not access deep flowers (Bartomeus et al. 2016). Thus, plant communities dominated by narrow or tubular flowers would be expected to support less diverse bee communities. Our results do not support this hypothesis. One possible explanation could be that there are multiple bee strategies to access deep flowers. For instance, body size and shape could also determine bee ability to exploit narrow flowers if oblong bees can enter these flowers to access nectar, despite their short mouthparts (Spaethe et al. 2001). Moreover, some wild bee species (e.g. bumble bees such as *Bombus terrestris*, *Bombus lapidarius*, *Bombus lucorum*) can rob nectar from flower buds by making holes near the base of the perianth of tubular flowers, which otherwise would not be accessible to them owing to their short tongues (Sáez et al. 2017).

### Grassland-to-crop conversion has a lasting impact on the relationship between plants and bees

Grassland-to-crop conversion modulated the functional linkage between plant and bee communities (Figs. 2 and 3b). The positive correlation between flower and bee trait diversity was not observed in landscapes where grasslands were converted into annual crops long ago compared to landscapes where grasslands have been only recently converted (Fig. 3b). Therefore, our results suggest that bee communities experienced time-delayed responses to habitat loss, consistently with our hypotheses. Decreasing grassland cover in agricultural landscapes has been shown to affect plant and pollinator communities (e.g. Clough et al. 2014). By considering land-use history, our study goes one step further and reveals that the effects of landscape simplification—through the decrease of grassland cover over time—are not only immediate but have also a lasting and negative impact on bee diversity. Our results highlight that land-use history could be a major driver of the disruption of plant–pollinator interactions currently observed, that may precede the loss of plant and pollinator species in agricultural landscapes (Valiente-Banuet et al. 2015).

Additionally, the local disturbance regime that occurred within grasslands directly modified the diversity of plants and bees, as illustrated by the positive effect of the age of the focal grassland on the flower (Fig. 1) and bee (Figs. 2, and 3c) trait diversity. Conversely to flower trait diversity, bee trait diversity flattened in 10 to 15 year-old grassland fields. Habitat stability over time is a key contributing factor to functional diversity in agricultural landscapes that can allow specialist species with low dispersal abilities to persist in intensive landscapes (Mouquet et al. 2003; Le Provost et al. 2017, 2020). We also found a linear positive effect of the proportion of grassland cover itself in the surrounding current landscape on bee trait diversity (Fig. 2). The presence of grasslands in the surrounding landscape can enhance bee trait diversity within local communities, independently of the local flower community, by creating landscape-scale spillover sustaining a flow of functionally contrasted species across bee communities (Blitzer et al. 2012).

### Impact of mass-flowering crop cover on the relationship between plants and bees

The positive relationship between plant and bee morphological diversities was also dependent on the proportion of mass-flowering crops in the surrounding landscapes. The positive effect of flower trait diversity on bee trait diversity weakened in landscapes dominated by a high cover of mass-flowering crops (Fig. 3a). Mass-flowering crops such as oilseed rape and sunflower are mostly visited by generalist pollinators, and increasing mass-flowering crop cover in the landscape benefits few dominant species sharing specific traits (Diekötter et al. 2010). After the cessation of the flowering, these generalist species may forage in the surrounding natural or semi-natural habitats, where resources are still available. Such landscape-level spillover can modify the trait distribution within these habitats, and locally distort the trait matching between plant and bee communities. Our data were collected in late summer, few months after the mass-bloom of oilseed rape and sunflower, which allowed us to specifically test how the lasting effect of mass-flowering crops in the landscape can impact the relationship between plant and bee trait diversities in grasslands. This result echoes previous studies which found that a high proportion of mass-flowering crops in the surrounding landscape disrupts local plant–pollinator interactions (e.g. Diekötter et al. 2010), which can in turn affect pollinators later in the year (Riedinger et al. 2014).

### Assessing bee trait diversity under contrasted land-use managements

There is great expectation that current strategies, aiming at enhancing floral resources at both local and landscape scales, will mitigate bee decline (e.g. agri-environmental scheme such as sowing nectar flower mixtures) (Scheper et al. 2014). When quantifying how bee diversity varied under contrasted land-use managements typically observed in our study region, we found that the persistence of functionally diverse bee communities is predicted to strongly decline in historically crop-dominated landscapes, even in flower-rich grasslands (Fig. 4). However, in real-world landscapes, different local and landscape drivers associated with current and past land-use changes may affect present-day biodiversity. By disentangling the effects of past and current land use operating at contrasted spatial scales (from the field- to the landscape-scale), our study may help to better understand the ultimate drivers of bee decline, and to refine conservation strategies. Consistently, our results indicate that a high proportion of grasslands in the surrounding landscape enhances local bee trait diversity, and that grasslands with high flower diversity support functionally diverse bee communities (Fig. 2). In agricultural landscapes, these grasslands may be more attractive than grasslands composed of less diverse plant communities and, by providing a wide array of feeding resources, could concentrate diverse bee communities (Clough et al. 2014). However, bee trait diversity in permanent and flower-rich grasslands strongly decreased in historically crop-dominated landscapes where grassland-to-crop conversion occurred long ago, compared to landscapes where grassland-to-crop conversion occurred only recently (Fig. 3). Past land-use changes may have led to an extinction debt within pollinator communities, and many pollinator species might be already extinct in agricultural landscapes (Kuussaari et al. 2009). While sowing grasslands in historically crop-dominated landscapes might help to avert further pollinator decline, restoring the past pollinator communities might therefore not be possible. Our study emphasized the urgent need to sanctuarise significant amount of permanent grasslands in agricultural areas, and to develop management strategies that ensure the sustainability of flower-rich grasslands to avoid future plant and pollinator loss (Bommarco et al. 2014).

To test the legacy effect of habitat loss, our study was based on a selection of grassland fields characterised by varying land-use change histories. This observational set up could be complemented by future long-term studies to explicitly monitor the time-delayed effects of past land-use changes on plant–bee interactions and the induced biodiversity loss. Such long-term studies can help to accurately predict the shape of pollinator response following land-use changes (e.g. linear or exponential decay), and to determine if restoration efforts could be implemented to avert further pollinator decline. Furthermore, we collected data at a specific period of the year (late summer) after the bloom of mass-flowering crops, in order to specifically test how current and past land use affect wild plant and bee communities in grasslands. However, bee phenology can modify the community composition over the growing season, and we might have under-estimated the importance of bee species that are more active in spring and early summer in driving bee response to land-use changes. Studies considering plant and bee communities at different times of the year could provide valuable insights on how land-use changes influence plant–pollinator temporal mismatch.

## Conclusions

Our study highlights the role of past habitat loss in shaping the present-day trait diversity of bee communities in agricultural landscapes. By considering landscapes characterized by different land-use histories, we show that past habitat degradation in agricultural landscapes can disrupt plant–bee interactions. The persistence of functionally diverse bee communities is predicted to strongly decline over time with the conversion of permanent grasslands into intensive annual crops. Therefore, plant and bee communities in agricultural landscapes may suffer strong extinction debts. This time-delayed response to habitat loss constitute an opportunity to avert further bee decline in farmlands, by developing long-term conservation and restoration efforts to preserve plant–bee mutualism. Long-term monitoring can help to further investigate the temporal dynamics of pollinator decline, to quantify the time needed to recover after disturbances, and to identify the ecological conditions that secure future pollination service in agricultural landscapes.

## Electronic supplementary material

Below is the link to the electronic supplementary material.

Electronic supplementary material 1 (DOCX 756 kb)

## References

[CR1] Aguilar R, Ashworth L, Galetto L, Aizen MA (2006). Plant reproductive susceptibility to habitat fragmentation: review and synthesis through a meta-analysis. Ecol Lett.

[CR2] Bailey S, Requier F, Nusillard B, Roberts SPM, Potts SG, Bouget G (2014). Distance from forest edge affects bee pollinators in oilseed rape fields. Ecol Evol.

[CR3] Bartomeus I, Gravel D, Tylianakis JM, Aizen MA, Dickie IA, Bernard‐Verdier M (2016). A common framework for identifying linkage rules across different types of interactions. Funct Ecol.

[CR4] Bartoń K (2014) MuMIn: Model selection and model averaging based on information criteria (AICc and alike). R Package Version 1–1

[CR5] Baude M, Kunin WE, Boatman ND, Conyers S, Davies N, Gillespie MAK, Morton RD, Smart SM, Memmott J (2016). Historical nectar assessment reveals the fall and rise of floral resources in Britain. Nature.

[CR6] Blitzer EJ, Dormann CF, Holzschuh A, Klein AM, Rand TA, Tscharntke T (2012). Spillover of functionally important organisms between managed and natural habitats. Agric Ecosyst Environ.

[CR7] Blonder B, Lamanna C, Violle C, Enquist BJ (2014). The n-dimensional hypervolume. Glob Ecol Biogeogr.

[CR8] Blonder B, Morrow CB, Maitner B, Harris DJ, Lamanna C, Violle C, Enquist BJ, Kerkhoff AJ (2018). New approaches for delineating n-dimensional hypervolumes. Methods Ecol Evol.

[CR9] Bommarco R, Lindborg R, Marini L, Öckinger E (2014). Extinction debt for plants and flower-visiting insects in landscapes with contrasting land use history. Divers Distrib.

[CR10] Borrell BJ (2005). Long tongues and loose niches: evolution of Euglossine Bees and their nectar flowers. Biotropica.

[CR11] Bretagnolle V, Berthet E, Gross N, Gauffre B, Plumejeaud C, Houte S, Badenhausser I, Monceau K, Allier F, Monestiez P, Gaba S (2018). Towards sustainable and multifunctional agriculture in farmland landscapes: lessons from the integrative approach of a French LTSER platform. Sci Total Environ.

[CR12] Bukovinszky T, Verheijen J, Zwerver S, Klop E, Biesmeijer JC, Wäckers FL, Prins HHT, Kleijn D (2017). Exploring the relationships between landscape complexity, wild bee species richness and reproduction, and pollination services along a complexity gradient in the Netherlands. Biol Conserv.

[CR13] Burkle LA, Alarcón R (2011). The future of plant–pollinator diversity: understanding interaction networks across time, space, and global change. Am J Bot.

[CR14] Campbell DR, Forster M, Bischoff M (2014). Selection of trait combinations through bee and fly visitation to flowers of Polemonium foliosissimum. J Evol Biol.

[CR15] Cariveau DP, Nayak GK, Bartomeus I, Zientek J, Ascher JS, Gibbs J, Winfree R (2016). The allometry of bee proboscis length and its uses in ecology. PLoS ONE.

[CR16] Clough Y, Ekroos J, Báldi A, Batáry P, Bommarco R, Gross N, Holzschuh A, Hopfenmüller S, Knop E, Kuussaari M, Lindborg R, Marini L, Öckinger E, Potts SG, Pöyry J, Roberts SPM, Steffan‐Dewenter I, Smith HG (2014). Density of insect-pollinated grassland plants decreases with increasing surrounding land-use intensity. Ecol Lett.

[CR17] Cornwell WK, Schwilk DW, Ackerly DD (2006). A trait-based test for habitat filtering: convex hull volume. Ecology.

[CR18] Coutinho JG, da Garibaldi E, Viana LA (2018). The influence of local and landscape scale on single response traits in bees: a meta-analysis. Agric Ecosyst Environ.

[CR19] Davis ES, Kelly R, Maggs CA, Stout JC (2018). Contrasting impacts of highly invasive plant species on flower-visiting insect communities. Biodivers Conserv.

[CR20] Deraison H, Badenhausser I, Börger L, Gross N (2015). Herbivore effect traits and their impact on plant community biomass: an experimental test using grasshoppers. Funct Ecol.

[CR21] Devictor V, Clavel J, Julliard R, Lavergne S, Mouillot D, Thuiller W, Venail P, Villeger S, Mouquet N (2010). Defining and measuring ecological specialization. J Appl Ecol.

[CR22] Diekötter T, Kadoya T, Peter F, Wolters V, Jauker F (2010). Oilseed rape crops distort plant–pollinator interactions. J Appl Ecol.

[CR23] Fontaine C, Dajoz I, Meriguet J, Loreau M (2006). Functional diversity of plant-pollinator interaction webs enhances the persistence of plant communities. PLoS Biol.

[CR24] Forrest JR, Thorp RW, Kremen C, Williams NM (2015). Contrasting patterns in species and functional-trait diversity of bees in an agricultural landscape. J Appl Ecol.

[CR25] Foster DJ, Cartar RV (2011). Wing wear affects wing use and choice of floral density in foraging bumble bees. Behav Ecol.

[CR26] García-Palacios P, Gross N, Gaitán J, Maestre FT (2018). Climate mediates the biodiversity–ecosystem stability relationship globally. Proc Natl Acad Sci.

[CR27] Garnier E, Cortez J, Billès G, Navas ML, Roumet C, Debussche M, Laurent G, Blanchard A, Aubry D, Bellmann A, Neill C, Toussaint JP (2004). Plant functional markers capture ecosystem properties during secondary succession. Ecology.

[CR28] Goulson D, Nicholls E, Botías C, Rotheray EL (2015). Bee declines driven by combined stress from parasites, pesticides, and lack of flowers. Science.

[CR29] Gravel D, Albouy C, Thuiller W (2016). The meaning of functional trait composition of food webs for ecosystem functioning. Phil Trans R Soc B.

[CR30] Greenleaf SS, Williams NM, Winfree R, Kremen C (2007). Bee foraging ranges and their relationship to body size. Oecologia.

[CR31] Hall M (2018). Blue and yellow vane traps differ in their sampling effectiveness for wild bees in both open and wooded habitats. Agric For Entomol.

[CR33] Holzschuh A, Steffan-Dewenter I, Kleijn D, Tscharntke T (2007). Diversity of flower-visiting bees in cereal fields: effects of farming system, landscape composition and regional context. J Appl Ecol.

[CR32] Holzschuh A, Dainese M, González-Varo JP, Mudri‐Stojnić S, Riedinger V, Rundlöf M, Scheper J, Wickens JB, Wickens VJ, Bommarco R, Kleijn D, Potts SG, Roberts SPM, Smith HG, Vilà M, Vujić A, Steffan‐Dewenter I (2016). Mass-flowering crops dilute pollinator abundance in agricultural landscapes across Europe. Ecol Lett.

[CR34] Klumpers SGT, Stang M, Klinkhamer PGL (2019). Foraging efficiency and size matching in a plant-pollinator community: the importance of sugar content and tongue length. Ecol Lett.

[CR35] Kremen C, Williams NM, Aizen MA, Gemmill-Herren B, LeBuhn G, Minckley R, Packer L, Potts SG, Roulston T, Steffan-Dewenter I, Vázquez DP, Winfree R, Adams L, Crone EE, Greenleaf SS, Keitt TH, Klein AM, Regetz J, Ricketts TH (2007). Pollination and other ecosystem services produced by mobile organisms: a conceptual framework for the effects of land-use change. Ecol Lett.

[CR36] Kuussaari M, Bommarco R, Heikkinen RK, Helm A, Krauss J, Lindborg R, Öckinger E, Pärtel M, Pino J, Rodà F, Stefanescu C, Teder T, Zobel M, Steffan-Dewenter I (2009). Extinction debt: a challenge for biodiversity conservation. Trends Ecol Evol.

[CR37] Larsen TH, Williams NM, Kremen C (2005). Extinction order and altered community structure rapidly disrupt ecosystem functioning. Ecol Lett.

[CR38] Lavorel S, Storkey J, Bardgett RD, de Bello F, Berg MP, Le Roux X, Moretti M, Mulder C, Pakeman RJ, Díaz S, Harrington R (2013). A novel framework for linking functional diversity of plants with other trophic levels for the quantification of ecosystem services. J Veg Sci.

[CR39] Le Bagousse-Pinguet Y, Soliveres S, Gross N, Torices R, Berdugo M, Maestre FT (2019). Phylogenetic, functional, and taxonomic richness have both positive and negative effects on ecosystem multifunctionality. Proc Natl Acad Sci.

[CR40] Le Provost G, Gross N, Börger L, Deraison H, Roncoroni M, Badenhausser I (2017). Trait-matching and mass effect determine the functional response of herbivore communities to land-use intensification. Funct Ecol.

[CR41] Le Provost G, Badenhausser I, Le Bagousse-Pinguet Y, Clough Y, Henckel L, Violle C, Bretagnolle V, Roncoroni M, Manning P, Gross N (2020). Land-use history impacts functional diversity across multiple trophic groups. Proc Natl Acad Sci.

[CR42] Martin EA, Feit B, Requier F (2019). Assessing the resilience of biodiversity-driven functions in agroecosystems under environmental change. Adv Ecol Res.

[CR43] McKinney ML, Lockwood JL (1999). Biotic homogenization: a few winners replacing many losers in the next mass extinction. Trends Ecol Evol.

[CR44] Mouquet N, Munguia P, Kneitel JM, Miller TE (2003). Community assembly time and the relationship between local and regional species richness. Oikos.

[CR45] Potts SG, Biesmeijer JC, Kremen C, Neumann P, Schweiger O, Kunin WE (2010). Global pollinator declines: trends, impacts and drivers. Trends Ecol Evol.

[CR46] Purschke O, Sykes MT, Poschlod P, Michalski SG, Römermann C, Durka W, Kühn I, Prentice HC (2014). Interactive effects of landscape history and current management on dispersal trait diversity in grassland plant communities. J Ecol.

[CR47] Requier F, Garnery L, Kohl PL, Njovu HK, Pirk CW, Crewe RM, Steffan-Dewenter I (2019). The conservation of native honey bees is crucial. Trends Ecol Evol.

[CR48] Requier F, Paillet Y, Laroche F, Rutschmann B, Zhang J, Lombardi F, Svoboda M, Steffan‐Dewenter I (2019b) Contribution of European forests to safeguard wild honeybee populations. Conserv Lett e12693

[CR50] Riedinger V, Renner M, Rundlöf M, Steffan-Dewenter I, Holzschuh A (2014). Early mass-flowering crops mitigate pollinator dilution in late-flowering crops. Landsc Ecol.

[CR51] Rollin O, Bretagnolle V, Decourtye A, Aptel J, Michel N, Vaissière BE, Henry M (2013). Differences of floral resource use between honey bees and wild bees in an intensive farming system. Agric Ecosyst Environ.

[CR52] Rollin O, Bretagnolle V, Fortel L, Guilbaud L, Henry M (2015). Habitat, spatial and temporal drivers of diversity patterns in a wild bee assemblage. Biodivers Conserv.

[CR53] Sáez A, Morales CL, Garibaldi LA, Aizen MA (2017). Invasive bumble bees reduce nectar availability for honey bees by robbing raspberry flower buds. Basic Appl Ecol.

[CR54] Scheper J, Reemer M, van Kats R, Ozinga WA, van der Linden GT, Schaminée JH, Siepel H, Kleijn D (2014). Museum specimens reveal loss of pollen host plants as key factor driving wild bee decline in The Netherlands. Proc Natl Acad Sci.

[CR55] Schielzeth H (2010). Simple means to improve the interpretability of regression coefficients. Methods Ecol Evol.

[CR56] Sirami C, Gross N, Baillod AB, Bertrand C, Carrié R, Hass A, Henckel L, Miguet P, Vuillot C, Alignier A, Girard J, Batáry P, Clough Y, Violle C, Giralt D, Bota G, Badenhausser I, Lefebvre G, Gauffre B, Vialatte A, Calatayud F, Gil-Tena A, Tischendorf L, Mitchell S, Lindsay K, Georges R, Hilaire S, Recasens J, Solé-Senan XO, Robleño I, Bosch J, Barrientos JA, Ricarte A, Marcos-Garcia MA, Miñano J, Mathevet R, Gibon A, Baudry J, Balent G, Poulin B, Burel F, Tscharntke T, Bretagnolle V, Siriwardena G, Ouin A, Brotons L, Martin JL, Fahrig L (2019). Increasing crop heterogeneity enhances multitrophic diversity across agricultural regions. Proc Natl Acad Sci.

[CR57] Spaethe J, Tautz J, Chittka L (2001). Visual constraints in foraging bumblebees: flower size and color affect search time and flight behavior. Proc Natl Acad Sci.

[CR60] Suding KN, Lavorel S, Chapin FS, Cornelissen JHC, Díaz S, Garnier E, Goldberg D, Hooper DU, Jackson ST, Navas ML (2008). Scaling environmental change through the community-level: a trait-based response-and-effect framework for plants. Glob Change Biol.

[CR61] Valiente-Banuet A, Aizen MA, Alcántara JM, Arroyo J, Cocucci A, Galetti M, García MB, García D, Gómez JM, Jordano P, Medel R, Navarro L, Obeso JR, Oviedo R, Ramírez N, Rey PJ, Traveset A, Verdú M, Zamora R (2015). Beyond species loss: the extinction of ecological interactions in a changing world. Funct Ecol.

[CR62] Vanbergen AJ, Initiative the IP (2013). Threats to an ecosystem service: pressures on pollinators. Front Ecol Environ.

[CR63] Venables WN, Ripley BD (2002) Modern Applied Statistics with S, Fourth edition. Springer, New York. ISBN 0-387-95457-0, http://www.stats.ox.ac.uk/pub/MASS4/

[CR65] Westphal C, Steffan-Dewenter I, Tscharntke T (2003). Mass flowering crops enhance pollinator densities at a landscape scale. Ecol Lett.

[CR64] Westphal C, Bommarco R, Carré G, Lamborn E, Morison N, Petanidou T, Potts SG, Roberts SPM, Szentgyörgyi H, Tscheulin T, Vaissière BE, Woyciechowski M, Biesmeijer JC, Kunin WE, Settele J, Steffan-Dewenter I (2008). Measuring bee diversity in different European habitats and biogeographical regions. Ecol Monogr.

