# Supplementary material for: Grassland-to-crop conversion in agricultural landscapes has lasting impact on the trait diversity of bees
Source: Landsc Ecol. 2020 Oct 18;36(1):281–95. doi: 10.1007/s10980-020-01141-2 (PMC7810634; doi:10.1007/s10980-020-01141-2)
Supplement: Supplementary file 1 — Electronic supplementary material 1 (DOCX 756 kb) [file 10980_2020_1141_MOESM1_ESM.docx]

**Journal name:** Landscape Ecology

**Title:** Grassland-to-crop conversion in agricultural landscapes has lasting impact on the trait diversity of bees

Gaëtane Le Provost^1234^*****<https://orcid.org/0000-0002-1643-6023>, Isabelle Badenhausser^1235^<https://orcid.org/0000-0002-6919-8647>, Cyrille Violle^6^, Fabrice Requier^7^<https://orcid.org/0000-0003-1638-3141>, Marie D’Ottavio^1238^, Marilyn Roncoroni^123^, Louis Gross^1239^, Nicolas Gross^10^

^1^ Centre d’Etudes Biologiques de Chizé UMR 7372, CNRS, Université de La Rochelle, F-79360 Villiers en Bois, France.

^2^ INRAE, USC 1339, Centre d’Etudes Biologiques de Chizé UMR 7372, CNRS, Université de La Rochelle, F-79360 Villiers en Bois, France.

^3^ LTSER « Zone Atelier Plaine & Val de Sèvre », Centre d’Etudes Biologiques de Chizé UMR 7372, CNRS, Université de La Rochelle, F-79360 Villiers en Bois, France.

^4^ Senckenberg Biodiversity and Climate Research Centre SBIK-F, Senckenberg Gesellschaft für Naturforschung, D-60325 Frankfurt, Germany.

^5^ INRAE, Unité de Recherche Pluridisciplinaire Prairies Plantes Fourragères, F-86600 Lusignan, France.

^6^ UMR 5175 CEFE, Univ Montpellier, CNRS, EPHE, IRD, Univ Paul Valéry 3, F-34293 Montpellier, France

^7^ Evolution Génome Comportement et Ecologie, CNRS, IRD, Université Paris-Sud, Université Paris-Saclay, F-91190 Gif-sur-Yvette, Paris, France.

^8^ Laboratoire de Lutte Biologique, Département des sciences biologiques, Université du Québec à Montréal (UQAM), C.P. 8888, Succ. Centre-Ville, Montréal, QC, Canada.

^9^ INRAE, UR 0633, URZF Unité de Recherche Zoologie Forestière, F-45075 Orléans, France.

^10^ UCA, INRAE, VetAgro Sup, UMR Ecosystème Prairial, F-63000 Clermont-Ferrand, France.

*** Corresponding author:** Gaëtane Le Provost

email: [gaetaneleprovost@free.fr](mailto:gaetaneleprovost@free.fr)

SUPPLEMENTARY MATERIAL

**
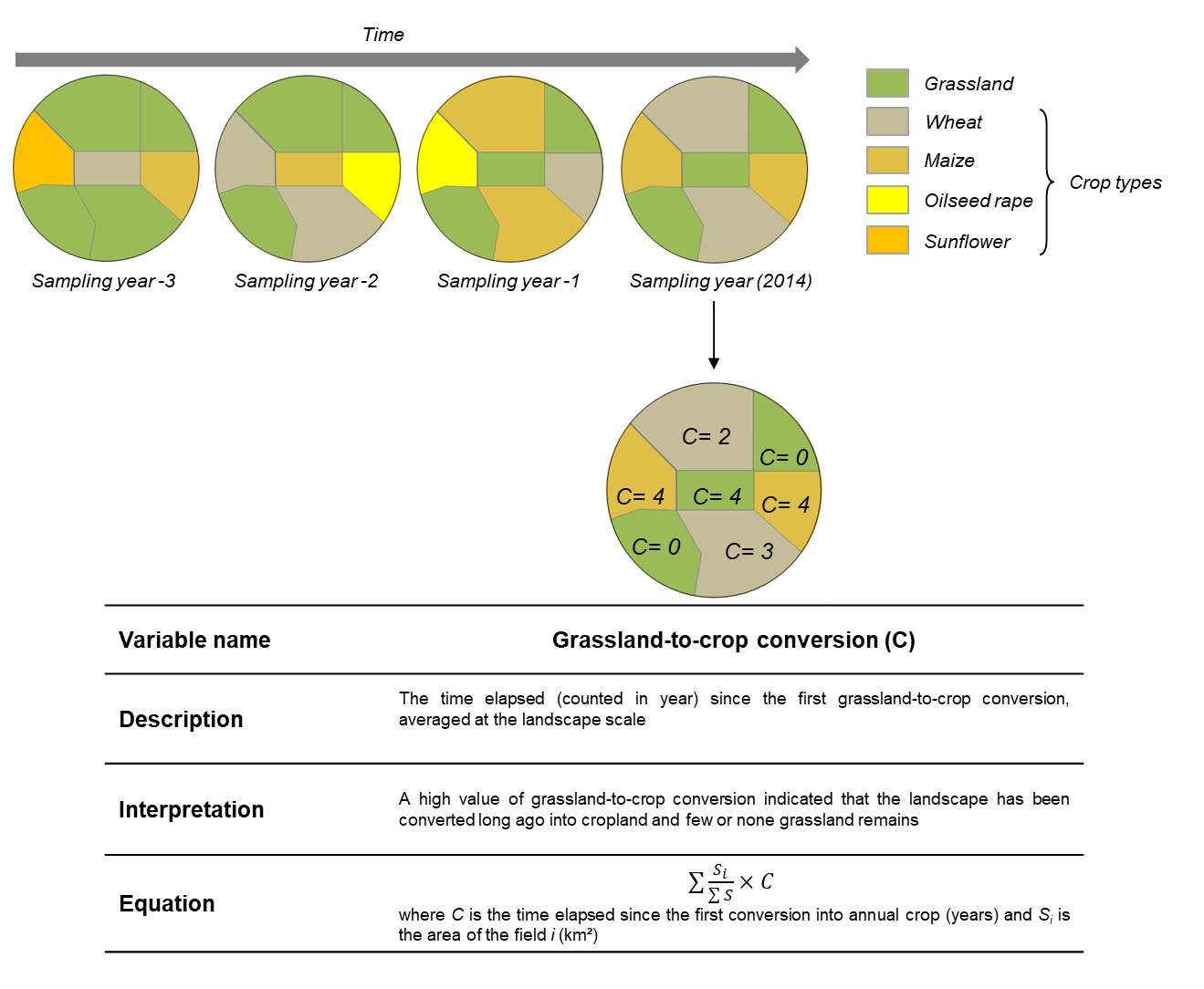
Fig. S1** Calculation of the metric quantifying the conversion of grasslands into annual crops in the landscape. The grassland-to-crop conversion metric (C) was calculated as the time elapsed (in year) since the first grassland-to-crop conversion for all fields in a 1 km-radius of the sampled grassland. We then weighted this metric by the field area (S) and averaged it for the entire 1-km landscape. This metric was calculated over 20 years (from 1994 to 2014). For simplicity, we illustrate the metric calculation with an example over 4 years. All information where extracted from our GIS database (QGIS v 2.14). In the study area, sown grasslands are often included in crop rotation. As they are regularly ploughed, the conversion from sown grassland to crop was not explicitly considered in our metric.

**Table S1** Correlations among all predictors included in the models (r)

|  | **Flower mean width** | **Flower mean depth** | **Flower mean ‘landing zone’** | **Age** | **Grassland productivity** | **% grassland** | **% forest** | **% mass-flowering** | **Grassland-to-crop conversion** | **X** | **Y** |
| --- | --- | --- | --- | --- | --- | --- | --- | --- | --- | --- | --- |
| **Floral hypervolume (log)** | -0.006 | -0.374 | 0.535 | 0.495 | -0.520 | 0.321 | 0.185 | -0.224 | -0.287 | -0.037 | -0.028 |
| **Flower mean width** |  | 0.567 | -0.007 | 0.209 | 0.180 | 0.258 | -0.161 | 0.163 | -0.047 | 0.132 | 0.225 |
| **Flower mean depth** |  |  | -0.160 | -0.276 | 0.125 | 0.115 | -0.275 | 0.152 | -0.146 | 0.072 | 0.279 |
| **Flower mean ‘landing zone’** |  |  |  | 0.400 | -0.248 | 0.202 | 0.049 | -0.260 | -0.216 | -0.182 | -0.087 |
| **Age** |  |  |  |  | -0.206 | 0.149 | 0.432 | -0.132 | -0.212 | -0.086 | -0.370 |
| **Grassland productivity** |  |  |  |  |  | -0.175 | -0.039 | 0.074 | 0.285 | 0.099 | -0.062 |
| **% grassland** |  |  |  |  |  |  | -0.106 | -0.395 | -0.480 | 0.162 | 0.044 |
| **% forest** |  |  |  |  |  |  |  | -0.279 | -0.181 | -0.154 | -0.549 |
| **% mass-flowering** |  |  |  |  |  |  |  |  | 0.373 | 0.261 | 0.174 |
| **Grassland-to-crop conversion** |  |  |  |  |  |  |  |  |  | 0.241 | 0.103 |
| **X** |  |  |  |  |  |  |  |  |  |  | 0.264 |

**
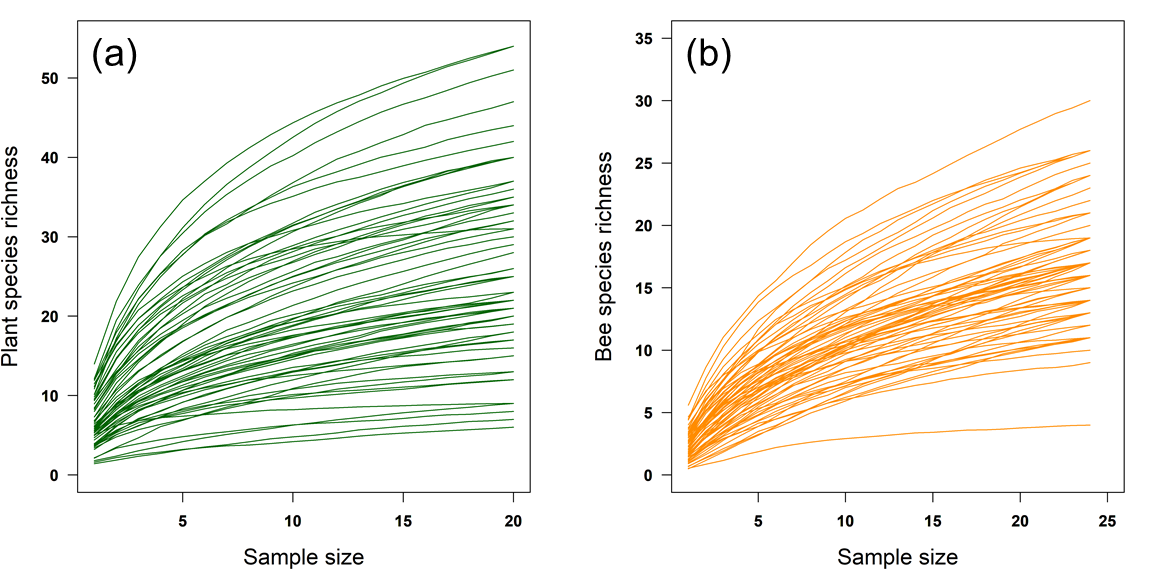
Fig. S2** Diversity accumulation curves for (a) plants and (b) bees based on the sampled grasslands and the sampling effort used. Lines correspond to the mean species richness detected depending on the sampling effort. Each line corresponds to a grassland field

**Table S2** Family and species names of the 25 plant species measured

| **Family** | **Species** |
| --- | --- |
| Asteraceae | *Bellis perennis* |
| Asteraceae | *Carduus sp* |
| Asteraceae | *Centaurea jaceae* |
| Caryophyllaceae | *Cerastium glomeratum* |
| Asteraceae | *Cirsium arvense* |
| Convolvulaceae | *Convolvulus arvensis* |
| Apiaceae | *Daucus carota* |
| Rubiaceae | *Galium mollugo* |
| Geraniaceae | *Geranium dissectum* |
| Fabaceae | *Hippocrepis comosa* |
| Asteraceae | *Leucanthemum vulgare* |
| Fabaceae | *Lotus corniculatus* |
| Fabaceae | *Medicago lupulina* |
| Fabaceae | *Medicago sativa* |
| Boraginaceae | *Myosotis arvensis* |
| Asteraceae | *Picris echioides* |
| Asteraceae | *Picris hieracioides* |
| Plantaginaceae | *Plantago lanceolata* |
| Renonculaceae | *Ranunculus acris* |
| Rosaceae | *Rubus fructosus* |
| Asteraceae | *Senecio jacobeae* |
| Asteraceae | *Senecio vulgaris* |
| Asteraceae | *Taraxacum officinalis* |
| Fabaceae | *Trifolium pratensis* |
| Fabaceae | *Trifolium repens* |
| Fabaceae | *Vicia sativa* |

| Family | Species |
| --- | --- |
| Andrenidae | *Andrena flavipes* |
| Andrenidae | *Andrena rosae* |
| Apidae | *Bombus lapidarius* |
| Apidae | *Bombus pascuorum* |
| Apidae | *Bombus sylvarum* |
| Apidae | *Bombus terrestris* |
| Apidae | *Ceratina cyanea* |
| Megachilidae | *Coelioxys inermis* |
| Melittidae | *Dasypoda hirtipes* |
| Halictidae | *Halictus maculatus* |
| Halictidae | *Halictus scabiosae* |
| Halictidae | *Halictus simplex* |
| Halictidae | *Halictus smaragdulus* |
| Halictidae | *Halictus subauratus* |
| Halictidae | *Halictus tumulorum* |
| Megachilidae | *Heriades truncorum* |
| Colletidae | *Hylaeus brevicornis* |
| Colletidae | *Hylaeus clypearis* |
| Colletidae | *Hylaeus communis* |
| Colletidae | *Hylaeus confusus* |
| Colletidae | *Hylaeus dilatatus* |
| Colletidae | *Hylaeus gibbus* |
| Colletidae | *Hylaeus pictipes* |
| Colletidae | *Hylaeus signatus* |
| Halictidae | *Lasioglossum calceatum* |
| Halictidae | *Lasioglossum clypeare* |
| Halictidae | *Lasioglossum fulvicorne* |
| Halictidae | *Lasioglossum glabriusculum* |
| Halictidae | *Lasioglossum griseolum* |
| Halictidae | *Lasioglossum interruptum* |
| Halictidae | *Lasioglossum laticeps* |
| Halictidae | *Lasioglossum lativentre* |
| Halictidae | *Lasioglossum leucozonium* |
| Halictidae | *Lasioglossum malachurum* |
| Halictidae | *Lasioglossum minutissimum* |
| Halictidae | *Lasioglossum minutulum* |
| Halictidae | *Lasioglossum morio* |
| Halictidae | *Lasioglossum nitidulum* |
| Halictidae | *Lasioglossum parvulum* |
| Halictidae | *Lasioglossum pauperatum* |
| Halictidae | *Lasioglossum pauxillum* |
| Halictidae | *Lasioglossum politum* |
| Halictidae | *Lasioglossum punctatissimum* |
| Halictidae | *Lasioglossum puncticolle* |
| Halictidae | *Lasioglossum subhirtum* |
| Halictidae | *Lasioglossum villosulum* |
| Halictidae | *Lasioglossum zonulum* |
| Megachilidae | *Lithurgus chrysurus* |
| Megachilidae | *Lithurgus cornutus* |
| Melittidae | *Melitta leporina* |
| Megachilidae | *Osmia spinulosa* |
| Andrenidae | *Panurgus dentipes* |
| Halictidae | *Sphecodes monilicornis* |
| Halictidae | *Sphecodes puncticeps* |
| Megachilidae | *Stelis breviuscula* |
| Megachilidae | *Stelis phaeoptera* |
| Megachilidae | *Stelis simillima* |
| Melittidae | *Tetralonia malvae* |
| Apidae | *Xylocopa iris* |
| Apidae | *Xylocopa violacea* |

**Table S3** Family and species names of the 60 wild bee species measured


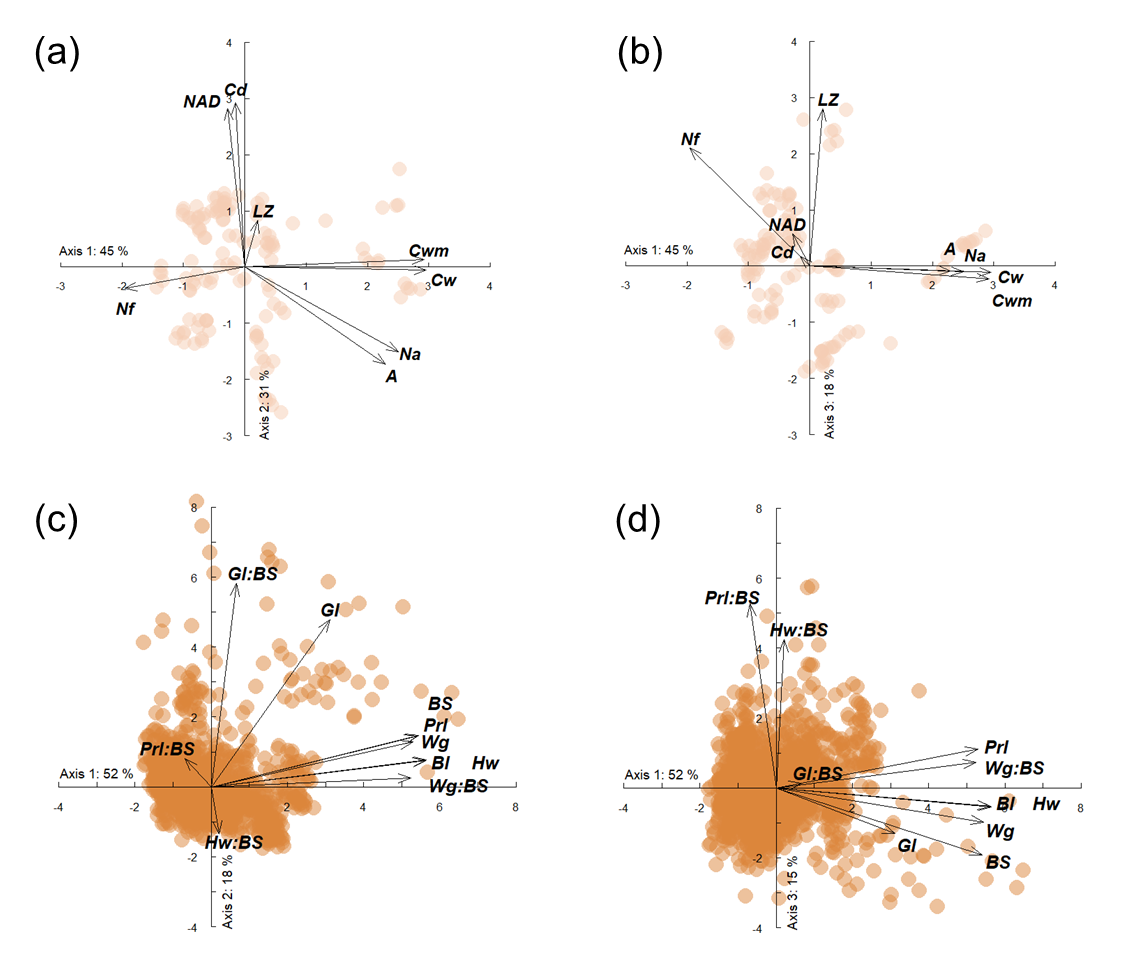
**Fig. S3** (a, and b) Floral traits, and (c, and d) bee morphological traits measured at the individual-level represented in two principal component analyses (PCA). The three orthogonal axes explained respectively 45 %, 31 % and 18 % of the total variance for floral traits, and 52 %, 18 % and 15 % of the total variance for bee traits. Floral traits segregated along three main axes of functional specialisation related to individual flower size, depth and inflorescence size (94 % of explained variance, see also Table S4). The first flower-PCA axis (45 % of explained variance) was positively correlated with the corolla diameter (Cw and Cwm), the nectar access (Na) and the flower-opening angle (A). The second flower-PCA axis (31 % of explained variance) was positively correlated with flower depth – corolla depth (Cd) and distance from the nectaries to the anthers (NAD). The third flower-PCA axis (18 % of variance explained) was positively correlated with the inflorescence diameter – landing zone (LZ) and the number of flowers (Nf). Bee traits segregated along three main bee-PCA axes related to size, glossa length and bee shape (85 % of explained variance, see also Table S5). The first bee-PCA axis (52 % of explained variance) was positively correlated with size- and foraging-related traits – body size (BS), body length (Bl), prementum length (Pl), head width (Hw), wing area (Wg) and the wing length *vs.* body size ratio (Wg:BS). The second bee-PCA axis (18 % of explained variance) was positively correlated with resource-acquisition traits - glossa length (Gl) and glossa length *vs.* body size ratio (Gl:BS). The third bee-PCA axis (15 % of explained variance) was related to bee shape (oblong individuals *vs*. thickset individuals) – positively correlated with prementum length *vs.* body size ratio (Prl:BS) and head width *vs.* body size ratio (Hw:BS).

**
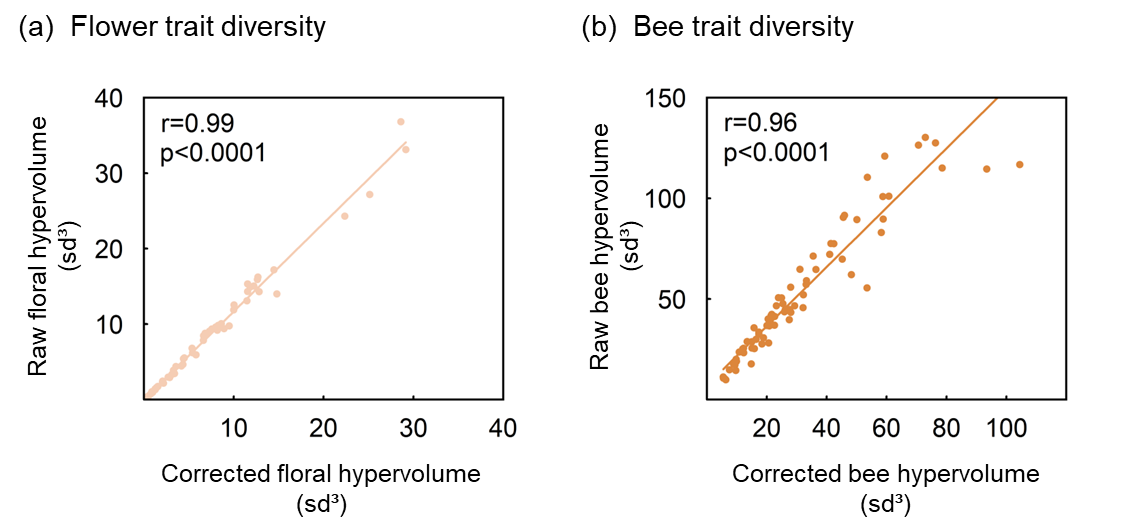
Fig. S4** Relationships between the raw hypervolume - calculated with all species or individuals sampled in the grassland - and the corrected hypervolume - calculated as the mean hypervolume of 100 samplings with a constant number of species or individuals draw from each (a) plant and (b) bee communities respectively

**Table S4** Correlations between plant flower traits and PCA axes (r). Bold coefficients indicated traits that are significantly associated with the PCA axis. See main text for trait abbreviations

| Traits | PCA Axis 1 | PCA Axis 2 | PCA Axis 3 |
| --- | --- | --- | --- |
| A | **0.764** | -0.576 | -0.027 |
| Cd | -0.050 | **0.977** | 0.058 |
| NAD | -0.094 | **0.942** | 0.191 |
| Nf | -0.652 | -0.128 | **0.699** |
| LZ | 0.072 | 0.276 | **0.932** |
| Na | **0.834** | -0.502 | -0.032 |
| Cw | **0.986** | -0.019 | -0.036 |
| Cwm | **0.972** | 0.045 | -0.076 |

**Table S5** Correlations between bee traits and PCA axes (r). Bold coefficients indicated traits that are significantly associated with the PCA axis. See main text for trait abbreviations

| Traits | PCA Axis 1 | PCA Axis 2 | PCA Axis 3 |
| --- | --- | --- | --- |
| BS | **0.900** | 0.242 | -0.319 |
| Bl | **0.936** | 0.124 | -0.088 |
| Wg | **0.907** | 0.245 | -0.163 |
| Hw | **0.941** | 0.128 | -0.091 |
| Wg:BS | **0.873** | 0.043 | 0.122 |
| Hw:BS | 0.035 | -0.222 | **0.707** |
| Gl | 0.519 | **0.797** | -0.217 |
| Prl | **0.881** | 0.214 | 0.185 |
| Gl:BS | 0.109 | **0.970** | 0.022 |
| Prl:BS | -0.115 | 0.136 | **0.877** |

**Table S6** Model selection table for flower trait diversity (TD). Colors correspond to different sets of predictor (yellow, current land-use variables; blue, land-use history variables; grey, geographic variables). Best models are presented ranked according to AICc value. Unshaded cells represent variables that were not included in the model

|  | Grassland productivity | % grassland | Grassland age | Y | R² | df | AICc | delta AICc | weight |
| --- | --- | --- | --- | --- | --- | --- | --- | --- | --- |
|  |  |  |  |  |  |  |  |  |  |
| Flower TD |  |  |  |  | 0.472 | 5.000 | 155.110 | 0.000 | 0.517 |
|  |  |  |  |  | 0.481 | 6.000 | 156.416 | 1.307 | 0.269 |
|  |  |  |  |  | 0.438 | 4.000 | 156.879 | 1.769 | 0.214 |

**Table S7** Best models selected from the multiple regressions for flower trait diversity

|  |  | **Flower trait diversity (hypervolume, sd^3^)** | | | | |
| --- | --- | --- | --- | --- | --- | --- |
|  | Model adjusted R² | 0.472 | | | | |
|  | Model parameters | Estimate | Std error | Std error adjusted | z value | P |
|  | Grassland productivity | -0.393 | 0.098 | 0.100 | 3.949 | **<0.001** |
|  | % grassland | 0.146 | 0.113 | 0.115 | 1.274 | 0.203 |
|  | Grassland age | 0.415 | 0.103 | 0.102 | 4.061 | **<0.001** |
|  | Y | 0.027 | 0.070 | 0.070 | 0.394 | 0.693 |

**Table S8** Model selection table for bee trait diversity (TD). Colours correspond to different sets of predictor (red, interactions ; pink, the functional structure of the flower community; yellow, current land-use variables; blue, land-use history variables; grey, geographic variables). Best models are presented ranked according to AICc value. Unshaded cells represent variables that were not included in the model

|  | Flower TD : grassland-to-crop conversion | Flower TD : % mass-flowering | Flower TD : % grassland | Mean landing zone | Mean flower depth | Mean flower width | Flower TD | % mass-flowering | | % grassland | Grassland age | | Grassland-to-crop conversion | | X | Y | R² | df | AICc | delta AICc | weight |
| --- | --- | --- | --- | --- | --- | --- | --- | --- | --- | --- | --- | --- | --- | --- | --- | --- | --- | --- | --- | --- | --- |
|  |  |  |  |  |  |  |  | X | X² |  | X | X² | X | X² |  |  |  |  |  |  |  |
| Bee TD (log) |  |  |  |  |  |  |  |  |  |  |  |  |  |  |  |  | 0.605 | 14.000 | -1.872 | 0.000 | 0.445 |
|  |  |  |  |  |  |  |  |  |  |  |  |  |  |  |  |  | 0.620 | 15.000 | -1.134 | 0.739 | 0.307 |
|  |  |  |  |  |  |  |  |  |  |  |  |  |  |  |  |  | 0.618 | 15.000 | -0.704 | 1.169 | 0.248 |

**Table S9** Best models selected from the multiple regressions for bee trait diversity (TD)

|  | **Bee trait diversity (log)** | | | | |
| --- | --- | --- | --- | --- | --- |
| Model adjusted R² | 0.605 | | | | |
| Model parameters | Estimate | Std error | Std error adjusted | z value | P |
| Grassland age | 0.184 | 0.051 | 0.052 | 3.516 | **<0.001** |
| Grassland age² | -0.110 | 0.027 | 0.028 | 3.964 | **<0.001** |
| Mean flower width | -0.095 | 0.040 | 0.041 | 2.312 | **0.021** |
| Mean flower depth | 0.228 | 0.042 | 0.043 | 5.377 | **<0.001** |
| Flower TD | 0.098 | 0.038 | 0.390 | 2.506 | **0.012** |
| % mass-flowering | -0.045 | 0.033 | 0.034 | 1.323 | 0.186 |
| % grassland | 0.082 | 0.036 | 0.037 | 2.208 | **0.027** |
| Grassland-to-crop conversion | -0.045 | 0.064 | 0.065 | 0.698 | 0.499 |
| Grassland-to-crop conversion² | -0.096 | 0.040 | 0.041 | 2.364 | **0.018** |
| Flower TD : % grassland | 0.009 | 0.025 | 0.025 | 0.367 | 0.714 |
| Flower TD : % mass-flowering | -0.109 | 0.033 | 0.034 | 3.189 | **0.001** |
| Flower TD : Grassland-to-crop conversion | -0.124 | 0.049 | 0.050 | 2.490 | **0.013** |
| X | -0.062 | 0.037 | 0.038 | 1.625 | 0.104 |
| Y | -0.077 | 0.034 | 0.035 | 2.176 | 0.030 |
